# A Systematic Review of the Safety, Feasibility and Benefits of Exercise for Patients with Advanced Cancer

**DOI:** 10.3390/cancers13174478

**Published:** 2021-09-06

**Authors:** Nico De Lazzari, Timo Niels, Mitra Tewes, Miriam Götte

**Affiliations:** 1 Department of Medical Oncology, West German Cancer Center, University Hospital Essen, 45147 Essen, Germany; Mitra.tewes@uk-essen.de; 2 Department I of Internal Medicine, Center of Integrated Oncology Aachen Bonn Cologne Düsseldorf, University Hospital of Cologne, 50937 Cologne, Germany; timo.niels@uk-koeln.de; 3 Department of Pediatric Hematology/Oncology, Clinic for Pediatrics 3, Center for Child and Adolescent Medicine, West German Cancer Center, University Hospital Essen, 45147 Essen, Germany; Miriam.goette@uk-essen.de

**Keywords:** advanced cancer, exercise, feasibility

## Abstract

**Simple Summary:**

Most advanced cancer patients suffer from severe symptoms due to cancer and medical treatment. Common symptoms are physical weakness, mental problems, and tiredness. Research has shown that exercise positively influences cancer-related side effects during and after treatment and longevity in cancer survivorship. However, exercise as a supportive therapy in advanced cancer patients is still not recommended in oncological guidelines. Therefore, the aim of this systematic review was to assess the safety, feasibility, and benefits of exercise for patients with advanced cancer. Based on the results of 14 included exercise intervention studies, we conclude that exercise is safe and feasible, seems to improve physical performance, and may lower symptoms like chronic tiredness. Early integration of exercise for advanced cancer patients should be considered as usual care as a supportive strategy.

**Abstract:**

Exercise therapy is a common supportive strategy in curative cancer treatment with strong evidence regarding its positive effects on, for example, cancer-related fatigue, health- related quality of life, and physical function. In the field of advanced cancer patients, knowledge about exercise as a useful supportive strategy is missing. The aim of this systematic review was to evaluate the feasibility and safety of exercise interventions as well as its effects on lowering the symptom burden. We included randomized controlled trials and nonrandomized controlled trials with advanced cancer patients receiving any type of exercise intervention. After an extensive literature search (in accordance to PRIMSA guidelines) in PubMed, Cochrane Library, and SPORTDiscus, 14 studies including 940 participants with different cancer entities were eligible. The results indicated the safety of exercise. In total, 493 participants received exercise interventions, with nine adverse events and no severe adverse events. The median recruitment rate was 68.33%, and adherence to exercise intervention was 86%. Further research with a high-quality and larger sample size is needed to clarify the potential of exercise with advanced cancer patients. Different advanced cancer entities have distinguished symptoms, and future research should construct entities-specific trial populations to figure out the best supportive exercise interventions.

## 1. Introduction

In Europe, 1.9 million deaths each year occur due to cancer [1]. Medical improvements achieved in the last years have led to longer survival periods in patients with incurable cancer [2,3]. Additional integration of palliative and supportive care results in a better quality of life and may prolong survival [3,4]. Even with limited life expectation, patients with advanced cancer can live several years. This highlights the importance of introducing interventions to raise patients’ quality of life and lower common side effects. However, a high percentage of advanced cancer patients (ACPs) suffer from a high symptom burden associated with high prevalence of pain (64%), fatigue (62%), anorexia (34%), constipation (32%), weakness (32%), and dyspnea (31%) [5,6,7,8,9], resulting in psychological distress and lowered quality of life. Further supportive care options are required to improve the quality of life and lower symptom burden. Exercise therapy is a promising method with the potential to decrease symptoms and treatment side effects in cancer patients and survivors. Accompanying exercise interventions during cancer treatment and survivorship received much attention in the last decades, with over 700 unique exercise trials with more than 50,000 cancer patients [10]. Current evidence displays the unique role of physical activity in reduction of cancer risk, beneficial prevention of different cancer entities, and improving longevity among cancer survivors. Evidence shows that regular physical activity is beneficial for the prevention of several types of cancer and for increased survival rates in cancer patients [11]. Furthermore, exercise therapy improves cancer-related fatigue, pain, dyspnea, quality of life, and physical fitness, and prevents muscle loss during active systematic treatment [12,13,14,15,16]. Especially, aerobic exercise is known to be an effective tool to manage cancer-related fatigue within early stage cancer and adjuvant treatment [17], and also improves cardiorespiratory fitness [18]. Resistance exercise within cancer patients improves lower limb and upper limb strength, muscle mass, and bone density [19,20]. In addition, resistance exercise during chemotherapy leads to higher completion rates [21]. Combined aerobic and resistance exercise can improve psychosocial health-related outcomes, e.g., anxiety and depression [22]. Exercise during chemotherapy or radiotherapy is also able to reduce harmful side effects of systematic treatment with decreased expression of symptoms [23,24,25]. However, most research on the therapeutical effects of exercise has been conducted during survivorship or during active cancer treatment of early stage cancer disease, leading to a large research gap in ACPs undergoing palliative treatment. Exercise therapy in incurable cancer patients is not yet approached as a standard recommendation [11,26]. This review aims to systematically summarize the evidence of exercise interventions with ACPs to support medical professionals, physiotherapists, and exercise therapist to develop evidence-based solutions to lower the symptom burden in ACPs. The last review on this topic was published by Heywood et al. in 2018 [27]. In addition to the evidence summarized in this 2018 publication, we also searched for the most recent studies from this rapidly evolving topic with the difference that only randomized (RCTs) and nonrandomized controlled trials (CTs) with 100% ACPs in trial were included. However, no review has analyzed different exercise modalities in exclusively ACPs with that strict inclusion criteria.

## 2. Materials and Methods

This systematic review was accomplished in accordance with the PRISMA guidelines [28], and the protocol was registered in PROSPERO database (CRD42020189850).

### 2.1. Information Sources and Searches

Sources for the conducted review were MEDLINE (PubMed), Cochrane Library, and EBSCO (SPORTDiscus). Searches were carried out on 10 July 2020. We performed an identical rerun on 16 June 2021. Main terms for the database search were as follows: ((neoplasms [MeSH] OR advanced cancer OR stage IV cancer Or incurable cancer OR metastatic cancer OR terminal cancer OR advanced tumor OR metastatic tumor OR terminal tumor OR incurable tumor OR stage IV tumor OR advanced tumour OR metastatic tumour OR terminal tumour OR incurable tumour OR stage IV tumour) AND (exercise [MeSH] OR aerobic exercise OR endurance exercise OR mobility exercise OR strength exercise OR exercise therapy OR aerobic training OR endurance training OR mobility training OR strength training OR training therapy) AND (feasibility OR fatigue OR quality of life OR strength OR endurance) AND (usual care OR palliative treatment)). Search terms were adapted to the respective databases. There were no restrictions regarding publication date. Written language of the publications was restricted to English or German, because of linguistic barriers of the authors.

### 2.2. Study Selection

Two independent authors (TN and NDL) screened databases for eligible articles. Studies were eligible if matching the following inclusion criteria: (a) randomized controlled trials (RCTs) or nonrandomized controlled trials (CTs); (b) human population ≥18 years with diagnosed advanced cancer (Stage III or IV); (c) exercise interventions involving endurance and/or resistance exercise; (d) control group received usual/standard care (no intervention) or physiotherapy, and (e) reported at least one of the following outcomes: primary outcome: feasibility of exercise intervention in ACPs based on adherence, drop-out rate, recruitment number, and adverse events; secondary outcomes: quality of life, fatigue, and physical performance assessed by objective measures, e.g., aerobic and strength tests. Combined interventions, e.g., nutrition support and exercise or psychosomatic and exercise, were excluded. Study abstracts and protocols were not eligible. After removing duplicates, two authors (TN and NDL) independently screened titles and abstracts. In case of disagreement for eligibility of titles and abstract or final inclusion, authors discussed until a consensus was reached. If no consensus could be reached, a senior author (MG) was contacted. Full text article screening of the eligible abstracts was conducted by (TN and NDL), accordingly. If full text articles did not contain sufficient data regarding relevant inclusion criteria (i.e., cancer stage), corresponding authors were contacted via email. The collecting of studies and screening were carried out with Citavi Version 6.0. (Swiss Academic Software GmbH, Zürich, Switzerland)

### 2.3. Outcome Assessment

As primary outcome, we evaluated the feasibility of exercise interventions in ACPs, which was assessed by adherence to intervention, drop-out rate, recruitment rate, and adverse events occurred during trial participation.

Secondary outcomes included questionnaire-based assessments for fatigue, quality of life, pain, sleep, and physical activity levels, and objective measurements of body composition, physical activity levels, and physical function.

### 2.4. Data Extraction and Quality Assessment

All data described in the protocol were extracted independently by two reviewers and inserted into a previously developed excel sheet. If the results were not matching, a consensus discussion between the two authors (TN and NDL) was carried out until final agreement. Extracted information included study type, study year, study population, sample size and participants’ demographics (age), outcomes and times of measurement, intervention type, duration, intensity and frequency of exercise, cancer type, stage of cancer, fatigue scores, feasibility measurement in form of adherence, drop-outs, recruitment rate, adverse events, outcomes of aerobic exercise, strength exercise, mobility exercise, and functional tests. Statistical variables extracted: mean, standard deviation, and *p*-values.

To determine the risk of bias in the randomized controlled trials, two authors (TN and NDL) independently assessed risk of bias for all included studies from five domains: Bias arising from randomization process, bias due to deviations from intended interventions, bias due to missing outcome data, bias in measurement of the outcome, and bias in selection of the reported result with the Cochrane risk of bias assessment (RoB 2.0, Cochrane, London, UK) [29]. Risk of bias assessment for clinical controlled trials (CCTs) was carried out by ROBINS-I tool (Cochrane, London, UK) [30] with seven bias domains (confounding, selection of participants, classification of interventions, bias due to deviations from intended interventions, missing data, measurement of outcomes, and selection of reported results). Disagreement was solved by discussion between two authors TN and NDL.

### 2.5. Data Syntheses and Statistical Analyses

After extracting the data of the included studies, a large heterogeneity was revealed with respect to included cancer types and measurements of the secondary effectiveness parameters due to different questionnaires or different assessments. This situation did not allow a meta-analysis of the existing studies.

## 3. Results

### 3.1. Characteristics of Included Trials and Participants

**Figure 1:**
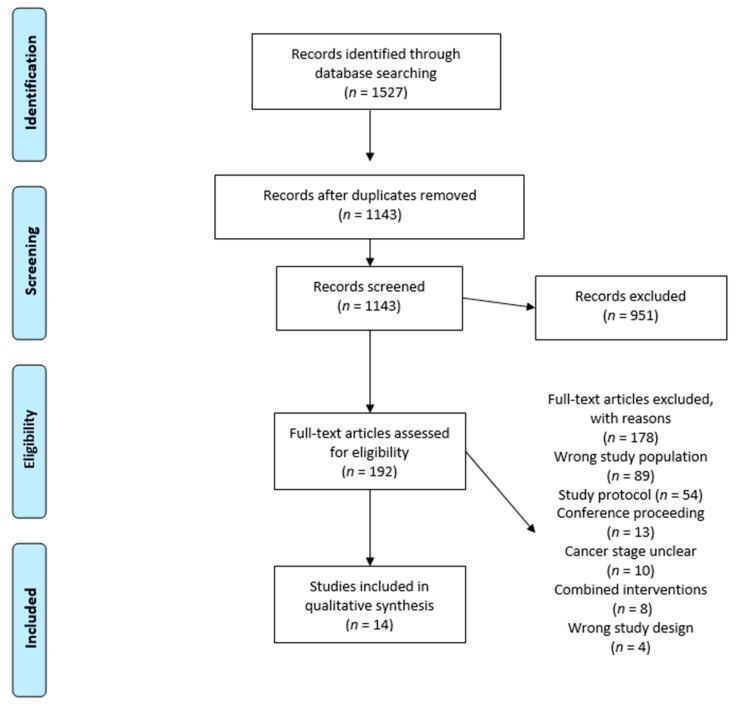
Preferred Reporting Items for Systematic Reviews and Meta-Analysis (PRISMA) flow diagram with number of studies identified and selected for inclusion in the systematic review [28]. Creative Commons Attribution License with permission if cited.

The database search resulted in *n* = 1327 records. After removing duplicates, *n* = 959 articles were screened for titles and abstracts, resulting in *n* = 186 records assessed for eligibility. After screening the full-text articles, *n* = 172 records were excluded (Figure 1: PRISMA flow diagram copyright by Cochrane group). Rerun search revealed *n* = 200 more eligible studies, which were screened for titles and abstracts, with *n* = 6 deemed eligible. Full-text screening exhibited no additional eligible studies. We contacted 12 authors due to insufficient information, with six responders, resulting in *n* = 14 records for inclusion. In total, 12 randomized controlled trials [31,32,33,34,35,36,37,38,39,40,41,42,43] and two controlled trials [44,45] were included, and the results are summarized in Table 1 and Table 2. In total, *n* = 940 patients were included, of which *n* = 493 patients received the exercise intervention and *n* = 447 patients formed the control groups. According to nine studies, patients were defined as ACPs by tumor stage IV, one study defined advanced after first line of treatment with palliative intent, one after clinical assessment, e.g., computed tomography, magnetic resonance imaging, and histological examination, and one study defined advanced with a short lifetime expectancy. Tumor entities were mixed in seven studies [31,32,33,36,40,41,43,44], two examined prostate cancer [35,38], two lung cancer [34,42], one nasopharyngeal cancer [37], one colorectal [39] and one investigated head and neck cancer [45]. Out of the 14 studies [31,32,33,34,35,36,37,38,39,40,41,42,43,44,45], 9 compared an exercise intervention with usual care [31,32,35,36,37,38,39,41,45], out of which *n* = 5 studies did not provide proper information about the actual meaning of usual care [32,37,40,42]. One study compared exercise to conventional physiotherapy [34], and one study compared exercise with usual care containing aspiration exercise and hot roll treatments [33,43].

### 3.2. Quality Assessment

**Figure 2:**
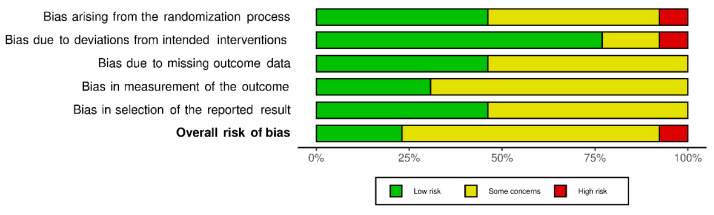

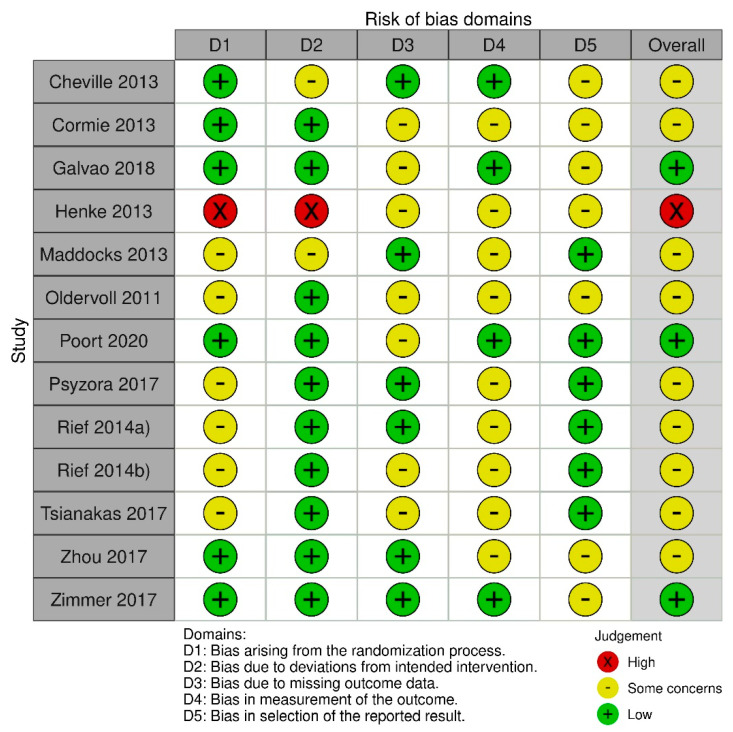
Risk of bias for randomized controlled trials (RoB 2.0).

**Figure 3:**
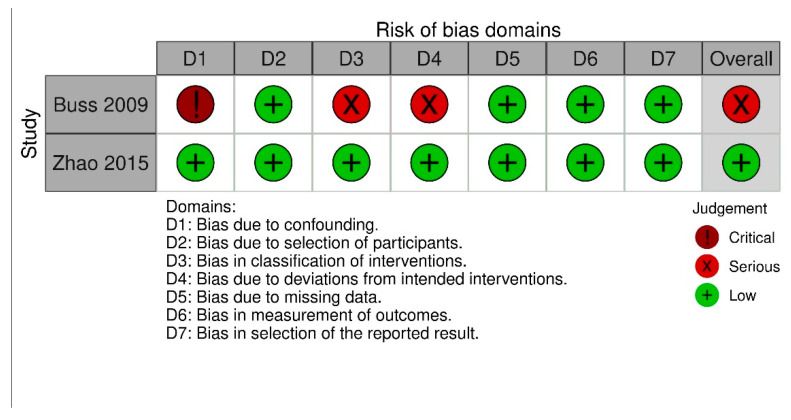

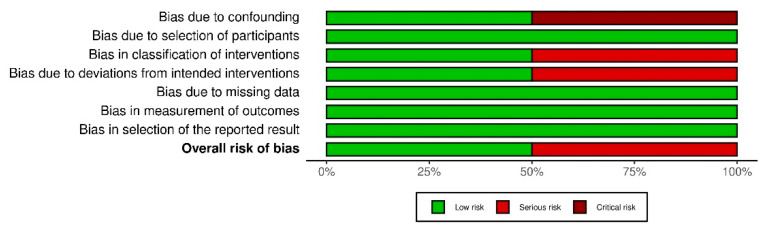
Risk of bias assessment for nonrandomized trials (ROBINS-I tool).

Quality assessment of the included studies is shown in Figure 2 and Figure 3. Overall, after assessment of RoB 2.0, three studies showed an overall low risk of bias [35,38,39], nine studies showed an overall rating with some concern [31,32,33,35,37,40,41,42,43], and one study was rated with a high risk [34]. This rating results from concerns with the randomization process and deviation from intended interventions. Two studies were assessed using the ROBINS-I tool, with one study having an overall low risk of bias [45]. A serious risk of bias was found in the second study [44], especially bias due to confounding, bias in classification of intervention, and bias due to deviations from intended intervention.

### 3.3. Intervention Description

Interventions in the observed studies mostly consisted of a combination of aerobic and strength exercise [31,32,33,36,38,39,45]. Two studies focused only on strength exercise [35,44], while two studies carried out aerobic walking exercise [40,41], one absolved Thai Chi exercise [37], one fulfilled kinesiotherapy exercise [44], and one focused on neuromuscular electrical stimulation (NMES) of the quadriceps [42]. Duration of intervention ranged from 2 weeks to 14 weeks. The intervention lasted for 12 weeks in four studies [35,36,38,40], followed by eight weeks in three studies [31,32,39], two studies completed a two-week training intervention [33,41,43], and one study duration was between 8 and 11 weeks (3–4 chemotherapy cycles). The longest study duration was 14 weeks [45], while one study exercised for 4 weeks [44] and one did not give proper information on the duration of the intervention [37]. Intensity of the exercise interventions was described precisely in three studies [34,36,39]. Strength exercise was applied from 50% to 80% of the maximal capacity (one-repetition maximum) with progressive increase. The intensity of aerobic exercise was precisely described in the same three studies [34,36,39]. Other studies [31,32,33,35,37,38,40,41,42,43,44,45] did not describe the intensity of the fulfilled interventions accurately. Exercise sessions per week varied between two times per week to seven times per week with different amounts of workload (active exercise minutes/week). Of the *n* = 14 [31,32,33,34,35,36,37,38,39,40,41,42,43,44,45] different interventions, *n* = 11 [32,34,35,36,37,38,39,41] were supervised exercise interventions. In one study, home-based training was performed [31]. Tsianakas et al. [40] conducted a group intervention supervised by volunteers. The participants of Maddocks et al. [42] completed the intervention in their own administration. Zhao et al. [45] and Rief et al. [33,43] completed a period of supervised training followed by home-based training.

### 3.4. Primary Outcome: Feasibility and Safety

The primary aim of the systematic review was to evaluate if structured exercise is feasible and safe in ACPs. Adverse events were defined as any injury that occurred related to exercise. Withdrawal from trial through disease-related reasons, for example, death due to cancer, was not considered as an adverse event related to exercise.

Recruitment rates were described in twelve studies [31,32,33,34,35,36,37,38,40,42,43,44,45], while two did not mention the screening and recruitment rates [39,41]. Overall, recruitment revealed a median of 68.3% (range: 25–87.6%). Four studies demonstrated a recruitment rate around 50% or lower [32,36,40,42]. Studies with a high recruitment rate (>60%) pointed out a low number of patients screened in the study conducted [31,33,34,35,43,45]. Barriers to recruitment were described in 12 studies [31,32,33,34,35,36,37,38,40,42,43,44,45]. In total, 544 declined trial participation. The most frequent reason for a nonparticipation was patients refused or declined (*n* = 269) participation. This was followed by health-related deterioration or conditions that were excluded because of the trial protocol (*n* = 82). Another reason for nonparticipation was the distance to study site. Overall, *n* = 45 patients declined because of travel distance, followed by *n* = 36 patients had no time for trial participation or were already actively involved in exercise (*n* = 14). However, there was also a big group of ACPs where the reasons were unknown (*n* = 76).

Adherence to exercise interventions was defined differently in studies. Five studies did not report adherence to exercise intervention [34,37,40,41,44], while eight studies did report adherence of exercise interventions with a median of 86% (range: 72–89%) [18,19,20,22,23,25,26,29,30,32]. Adherence changes between trial setting (e.g., supervised/unsupervised or home-based interventions) were not observed.

Overall, ten studies [31,32,35,36,38,39,40,42,45] reported adverse events as an outcome of measurement, while four studies [33,34,41,43,44] did not assess and report adverse events. In total, *n* = 940 patients were observed in the prescribed studies, with *n* = 220 (23.4%) drop-outs, *n* = 128 (58.1%) patients in the intervention groups, and *n* = 92 (41.8%) patients in control groups. The primary reason for dropping out was progression of cancer disease or health deterioration (*n* = 113), followed by death due to cancer (*n* = 61). All reasons for drop-outs are displayed in Table 3.

Two studies [36,42] reported adverse events, such as NMES-related muscle discomfort Grade 1 after CTC (*n* = 3) [42], muscle pain overuse (*n* = 4), cardiac complaints (*n* = 1), and increased fatigue (*n* = 1) [36]. Only three adverse events resulted in drop-outs, and all occurred in one study [33]. Of patients who received exercise therapy, *n* = 493 participants had nine exercise-related adverse events, considered at 2.01% of all patients who participated in exercise interventions. No further serious adverse events related to exercise were reported.

### 3.5. Secondary Outcomes

#### 3.5.1. Quality of Life

Overall QoL was assessed in ten studies [31,34,35,36,39,40,42,43,44,45], with a combined total of 470 patients included. Only two studies [39,43] revealed significant improvement of QoL, with a total sample size of 90 patients (EX = 47, C = 43). Five studies [34,40,42,44,45] with a combined total sample size of 214 patients (EX = 125, C = 89) demonstrated a decreased QoL in control group after intervention period while exercise group remained unchanged or slightly increased but without significant statistical values. In three studies [31,35,36], with a total of 166 patients included (EX = 77, C = 89), QoL remained stable/slightly increased in exercise and control groups without significant *p*-values.

#### 3.5.2. Fatigue

In total, thirteen studies [31,32,33,34,35,36,37,38,40,41,42,44,45] with a combined total sample size of 910 patients investigated the influence of exercise on fatigue. Improved fatigue was demonstrated in six studies [31,33,37,41,44,45], with a total of 377 patients observed (EX = 199, C = 178). No statistically relevant changes between groups were detected in six studies [23,25,26,27,29,31], with a total of 484 patients observed (EX = 247, C = 237). A worsening of fatigue was shown in one [42] trial with 49 participants (EX = 30, C = 19).

#### 3.5.3. Physical Function

Physical function was assessed in nine studies, whereof four studies [32,35,38,39] with a combined total of 338 participants (EX = 176, C = 162) revealed improvements in strength-related assessments (1RM, handgrip, bench press, or leg press).

One study [33] with 60 patients (EX = 30, C = 30) could prove significant improvement below the anaerobic threshold, while four studies [35,38,39,45] with a combined total of 100 patients (EX = 66, C = 61) displayed no significant changes. Aerobic exercise capacity improvement was detected in one study [35] with 20 patients (EX = 10, C = 10), while another one study [38] with 57 patients (EX = 28, C = 29) could not detect improvement.

#### 3.5.4. Regular Physical Activity Levels

Three studies [35,42,45] with a combined total of 89 patients (EX = 51, C = 38) measured regular physical activity levels. Improvements were noted in *n* = 1 study [35] with 20 patients (EX = 10, C = 10), while two studies [42,45] with a combined total sample size of 69 (EX = 41, C = 28) displayed no increased physical activity levels.

#### 3.5.5. Body Composition

Overall, four studies with a combined total of 166 patients (EX = 89, C = 77) assessed body composition before and after study participation [35,38,42,45]. Cormie et al. [35], with a sample size of 20 patients (EX = 10, C = 10), revealed significant improvements of lean body mass in the exercise group. No significant changes were displayed in two studies [38,45] with combined total sample size of 77 (EX = 39, C = 38). One study [33] with 49 patients (EX = 30, C = 19) evaluated deteriorations of body composition (lean body mass) in both groups after the trial period.

#### 3.5.6. Sleep

Only one study [31] with 66 patients (EX = 33, C = 33) assessed sleep, with a significant improvement demonstrated.

### 3.6. Effect According to Intervention

#### 3.6.1. Combined Aerobic and Strength Exercise

In total, nine studies [31,32,34,35,36,37,38,39,45] with a combined total of 672 patients (EX = 344, C = 328, combined endurance and strength training; *n* = 8 [31,32,34,35,37,38,39,45], with a total of 584 patients (EX = 302, C = 282), proved a significant improvement in some domains of physical performance (e.g., shuttle walk test, handgrip, six-minute walking test, bench press and leg press) (87.5%), and only one study with 66 patients (EX = 33, C = 33) found a significant difference in fatigue scores and sleep quality between exercise and usual care [31]. One study [36] with 88 patients (EX = 42, C = 46) did not see any improvements after combined exercise intervention. No improvements due to combined intervention could be found in quality of life.

#### 3.6.2. Single Aerobic Exercise

Only one study [40] with 42 patients (EX = 21, C = 21) completed only aerobic exercise, and it did not display any significant changes.

#### 3.6.3. Single Strength Exercise

Three RCTs [33,41,42] and one CT [44] with a combined total of 226 patients (EX = 128 C = 98) performed only strength-related exercise, with significant improvements in fatigue, fatigue subscales, and fatigue severity. One Study [33] with 60 patients (EX = 30, C = 30) also indicated improvements in quality of life due to strength exercise. One study [42] with a sample size of 49 (EX = 30, C = 19) displayed no significant changes of fatigue, quality of life, or physical performance compared to usual care.

#### 3.6.4. Home-Based Exercise

One study [31] with 66 patients (EX = 33, C = 33) carried out a full home-based intervention, with significant changes in fatigue, sleep, and mobility. Two studies [33,45] with a combined sample size of 80 patients (EX = 41, C = 39) performed a mixed trial setting, starting with supervised training and followed by a home-based period, with significant changes related to 30 s sit-to-stand, fatigue, pain, quality of life, vitality, and strength.

## 4. Discussion

This systematic review summarized the evidence on the feasibility of exercise interventions in ACPs. The results of the presented studies show that exercise interventions are feasible in terms of adverse events, recruitment rate, adherence to intervention, and drop-outs. Reported adverse events appear low grade and reflect common body reactions that might potentially be controlled by an improved training management. However, adverse events have to be considered with caution because grading of adverse events has not been provided. This is in accordance to other published studies related to adverse events while exercising with ACPs [46,47]. Future studies should aim for a detailed collection of adverse events with grading using common terminology criteria for adverse events (CTCAE v5.0 [48]).

Drop-out rates in trials with ACPs show a marked difference compared to trials with tumor patients in earlier stages. The drop-out rate in exercise interventions in a curative treatment setting was reported to be 10–13% [49,50]. Our aggregated data displayed drop-out rates of around 22%. In other supportive and palliative care trials, the drop-out-rates were 26% with similar reasons to those mentioned in the result section [51], i.e., expected drop-out because of disease progression and health deterioration. This seems to be valuable information when planning new research studies, e.g., for sample size calculations. To clearly identify the influence on the primary endpoint and avoid contamination, around 25% more patients should be included.

Adherence to exercise interventions was reported in nine of the included studies [31,32,33,35,36,38,39,42,45], while five studies [34,37,40,41,44] did not report adherence to intervention. The range of adherence was between 72% and 89%; however, the actual rate could have differed if adherence had been provided in all studies.

Median recruitment rates of exercise studies in the oncology field are 38% for colorectal cancer patients [52] and 59% for lung cancer patients in mixed stages I–IV [53]. The median enrollment rate of included studies of our review was 65.7%. Only two studies [32,36] screened more than 200 patients. Studies with a very specific study protocol, for example, studies of Cormie et al. [35] or Rief et al. [33,43], increased the median by screening only a small patient population. Overall, two studies [32,36] screened a high number of patients and pointed out recruitment rates of 68.3%. However, the recruitment process still shows barriers for advanced patients to participate in trials with exercise interventions. It is already well known that staying physically active during cancer treatment is a common challenge and sedentary behavior is present in a high proportion of patients [54]. There are several factors inhibiting patients from participating in exercise activities, even if patients consider exercise to be helpful for physical or psychosocial aspects [55,56]. Barriers to exercise are symptoms and treatment side effects, medical complications, comorbidities, confusion about the capability of exercise, and distance to study sites [57,58,59]. Physicians and supportive care teams need to identify these individual barriers and find solutions to overcome these to reduce nonparticipation.

However, these studies provide evidence that exercise can be beneficial for improvements or for stabilization of fatigue. Considering the sample size, trials with more than 50 participants [31,33,37,41] showed a significant reduction in fatigue. However, all of the studies raise concerns regarding the overall quality of assessment. A smaller study, e.g., the study of Zhao et al. [45], could also show significant improvement of fatigue in combination with overall low risk of bias. Other trials were not able to statistically demonstrate significant differences due to the small sample size. In contrast, the study by Oldervoll et al. [32] with a large study group of 231 patients showed no significant difference in fatigue. In view of the methodological evaluation carried out, the study cited reveals four domains of the RoB 2.0 as of concern. The authors also discuss reasons for not generating significant results as progression of the disease within the intervention period and a significantly increased baseline fatigue compared to other trials. Cancer-related fatigue affects almost all ACPs and correlates with the quality of life; high fatigue indicates a lower quality of life [60]. Furthermore, increased fatigue levels lead to physical inactivity and consequently, loss of physical performance and muscle mass [61]. The included studies conducted different interventional modalities (e.g., home-based, supervised, resistance exercise, combined resistance endurance exercise, Tai-Chi, intensity, duration, frequency) that influenced fatigue positively. Cancer-related fatigue is known to alter different biological pathways during the trajectory of cancer, such as muscle metabolism or pro-inflammatory cytokines, due to the tumor itself or as a treatment side effect [62,63]. Exercise can reverse this biological process and alleviate the expression of different biological pathways [64]. Further research should address which exercise principles are most beneficial. It remains unclear up to which point interventions against fatigue should be applied; the European Association for Palliative Care (EAPC) discusses that in the end-of-life stage, fatigue can also give a protection or shield [65]. Interventional treatment for fatigue could have a harmful on this protection and affect quality of life.

Considering the methodological quality of the provided studies and the sample size, it appears that exercise with ACPs can improve or at least stabilize QoL. The studies we rated with an overall low risk of bias [36,39] both showed an increase in QoL, although this difference was not significant in the study of Poort et al. [36]. Poort et al. had already mentioned the high drop-out in exercise group as a potential reason. Overall, the sample sizes of studies with QoL as an endpoint are mostly small, and evidence is limited regarding QoL as an endpoint.

Several studies underlined the positive effects of exercise on physical performance and function in ACPs [31,32,33,34,35,38,39,45]. Especially, studies with more than 50 participants [31,32,33,38] showed an improvement in physical domains. Furthermore, studies rated with a low overall risk of bias [38,39] also showed significant improvement despite the small sample size. Exercise in ACPs can significantly improve physical performance. Loss of physical function significantly correlates with low quality of life in ACPs [66]. Decline of physical function is a predictor for negative changes in mental components of health-related quality of life [67]. Therefore, exercise could be a considered a promising solution to improve physical function and improve associated patients’ related outcomes (PROMS). The intervention context was mostly supervised with combined strength and endurance units [32,33,34,35,38,45]. Trial settings with home-based parts seem to be promising as well and revealed positive results on PROMS. Three studies [31,32,43,45] solely included home-based interventions or mixed models with supervised sessions followed by home-based training. Home-based training seems to be impactful as well, and could be a future option to lower barriers to improve PROs. Multidimensional cancer rehabilitation delivered online in early cancer and survivorship had a high acceptability and also revealed significant improvements in physical function [68]. Macdonald et al., 2020, displayed a solid recruitment rate (64%) and impressive retention rate (83%) and adherence rates (80%). Future research should address the question of supervised vs. home-based training in ACPs. Over the last decades, the quantity of telemedicine-delivered interventions has increased rapidly, providing a promising opportunity for advanced cancer exercise trials. Home-based interventions delivered by telemedicine are proven to be feasible and safe [69,70]. The application of telemedicine could significantly reduce barriers of ACPs and reduce additional burdens of participation.

## 5. Limitations

The main weakness of the review is the high heterogeneity of the different studies. Meta-analysis could not be performed due to the different test procedures or questionnaires. Overall, studies have a small sample size, and some studies were unclear about the comparison to exercise and intensity of exercise that was completed by exercise participants. Risk of bias indicates some methodical variations in nearly all provided studies. Most of the described studies have some methodical concerns. Moreover, we did not search all possible literature sources for exercise studies in ACPs because of linguistic limitations. As a result, studies may be missing. However, exercise seems feasible, potent to increase physical performance, and may lower disease-specific symptoms, such as fatigue. Exercise is cost efficient and easy to apply as a supportive management tool. Patients who are working out actively have the opportunity to actively improve their living situation. This strengthens resilience against the life-shortening illnesses.

## 6. Conclusions

Regardless of the heterogeneous study situation within the evaluated trials, exercise interventions seem to be safe and feasible in ACPs. The summarized evidence indicates beneficial effects of exercise on physical performance and QoL, and a potential reduction of fatigue. The combination of aerobic and strength exercise seems preferable to improve physical function. Further research with complete reporting of the exercise adherence should examine these preliminary beneficial effects in multicenter studies to provide evidence regarding the ability of different exercise modalities to lower symptom burden and improve PROs. Based on our results, exercise should be offered to ACPs as well as people receiving curative treatment.

## Figures and Tables

**Table 1:** Study characteristics of randomized controlled trials.

Author	Cancer Entities	Age	Treat	*n* (EX/C)	Description of Intervention	Exercise Modalities	Control	Recruit-ment (Participants/Eligible)	Drop-Out Rate:	Adherence	AE	EX vs. UC ↑ = Improvement ↓ = Worsening	EX vs. UC (Non Sign.)
Cheville et al., 2013 [31]	Lung and colorectal cancer; Stage IV (*n* = 32 colon cancer; *n* = 34 lung cancer)	Ex: 63.8 ± 12.5; C: 65.5 ± 8.9	CTx RTx MAB	66 (33/33)	8-wk homebased REX and walking exercise	4x/wk: incremental Walking: 20 min, briskly, ~3.5 mets, REX: upper/lower body split, 5 exercise; 10–15 reps	Usual care	66/93: (70.9%)	EX: 7/33 (21%) C: 3/33 (9.9%), in total 10/66 (15.1%)	20/26 (76.9%) pts reached requirement for participation	0	↑ AM-PAC mobility (*p* = 0.02) ↑Fatigue (FACT-F) (*p* = 0.03) ↑ Sleep (NRS) (*p* = 0.05)	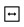 Ambulatory Post Acute Care Daily Activities Short Form (AM-PAC CAT) 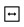 QoL (FACT-G)
Cormie et al., 2013 ∞ [35]	Prostate cancer; secondary bone metastases (*n* = 20)	Ex: 73.1 ± 7.5; C: 71.2 ± 6.9	Prev. AST RTx SX	20 (10/10)	12-wk suplow-level AEX and REX targeting major muscle groups	2x/wk; REX on machines; 8–12 reps, 2–4 sets, ~60 min, Low-level aerobix exercise and stretching	Usual care	20/27: (74%)	EX: 2/10 (20%) C: 3/10 (30%) in total 5/20 (25%)	83% attendance (mean: 20.2 ± 7.6 out of 24 sessions); 70% pts completing 24/24 sessions	0	↑ Physical function (*p* = 0.016) ↑ 400-m Walk (*p* = 0.010) ↑ 6 MWT; usual pace (*p* = 0.001) ↑ Body lean mass (*p* = 0.026) ↑ Lean mass (*p* = 0.003)	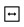 Fatigue 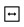 QoL
Galvao et al., 2018 [38]	Prostate cancer; bone metastases (*n* = 57)	Ex: 69.7 ± 7.6; C: 70.4 ± 9.3	AST CTx	57 (28/29)	12-wk sup, combined AEX, REX, and flexibility exercise	3x/wk: REX: progressively; 10–12 reps; 3 sets AEX: 20–30 min; Cycling/treadmill; 60–85% of max. HR, Flexibility EX: static stretching; 2–4 reps, each muscle group 30–60 s	Usual care	57/103: (55.3%)	EX: 5/28 (17.8) C: 3/29 (10.3%) in total 8/57 (14%)	Session: 32 ± 10/36; 89% attendance	0	↑ physical function (NBS) (*p* = 0.028) ↑ leg extension (*p* = 0.033)	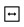 6 MWT 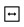 400 m walk test 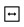 Up and Go Test 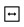 SOT 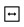 Lean mass 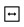 Body fat mass. 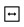 Fatigue (FACT-F)
Henke et al., 2013 [34]	Lung cancer Stage IIIA/IIIB/IV (*n* = 46)	n.a. *	CTx	46 (25/21)	3 chemotherapy cycles long: Sup Combined REX and AEX	7x/wk REX: resistance bands; 50% of max. capacity. 10 reps, 3 sets 5x/wk: AEX: 6 min walking moderate intensity (55–70% of HRmax)	Conventional physiotherapy	46/70: (65.7%)	EX: 6/24 (25%) C: 9/20 (45%) in total 15/44 (34%), *n* = 2 dropouts before randomization	n.a. *	n.a. *	↑ 6 MWT (*p* < 0.05) ↑ Staircase walking (*p* = 0.05) ↑ Physical functioning (C-30/LC-13, *p* = 0.025) ↑ Cognitive functioning (C-30/LC-13, *p* = 0.050	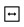 Global health status/QoL 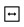 Role functioning (C-30) 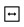 Emotional functioning (C-30) 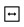 Symptom Scales (C-30/L-13)
Maddocks et al., 2013 [42]	NSCLC cancer; Stage IV (*n* = 49)	Ex: 70; C: 68, n.a. * STD	CTx	49 (30/19)	3–4 chemotherapy cycles long (8–11 wk) self-administered neuromuscular electrical stimulation (NMES) of the quadriceps Muscle	3–7x/wk; 30 min/session each leg; intensity: 50 Hz; frequency: 350 microseconds pulse width; duty cycle: 11–25%; 0–120 m)	n.a. *	49/190: (25%)	EX: 15/30 (50%) C: 6/19 (31.5%) in total 21/49 (42.8%)	9/15 completing at least 3x/wk	*n* = 3 NMES-related muscle discomfort resulting in dropout (CTC grade I)	↑ Mental Fatigue (*p* = 0.03)	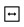 Quadriceps muscle strength 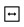 Thigh lean mass 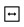 Whole Body lean mass 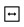 Step count 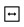 Fatigue 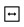 QoL
Oldervoll et al., 2011 [32]	Incurable, metastatic cancer, and life expectancy of 3–24 months (*n* = 41 gastrointestinal, *n* = 23 breast, *n* = 21 lung, *n* = 16 urological, *n* = 8 gynaecological, *n* = 2 hematological, and *n* = 10 other cancer)	Ex: 62.6 ± 11.3; C: 62.2 ± 10.7	CTx RTx HT TT	231 (121/110)	8-wk sup, combined exercise	2x/wk; 50–60 min, AEX: Warmup 10–15 min + Circuit EX: 6-station circuit; 2 min on, 1min off, combining strength, aerobic, and coordination elements.	n.a.*	231/400: (57.7%)	EX: 43/121 (35.5%) C: 25/110 (22.7%) in total 68/231 (29.4%)	69% finished pre and post assessment sessions: 11/16	0	↑ Shuttle walk test (*p* = 0.008) ↑ Handgrip strength (*p* = 0.01)	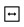 Total fatigue 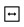 Physical fatigue 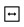 Mental fatigue 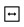 Sit-to-stand
Poort et al., 2020 [36]	Cancer patients with palliative systemic treatment (*n* = 35 breast, *n* = 21 colorectal, *n* = 19 prostate, *n* = 4 renal cell, *n* = 4 ovarian, *n* = 4 melanoma, *n* = 1 bladder cancer)	Ex: 60,67 ± 10.7; C: 63.9 ± 8.9	CTx HT TT CTx + TT HT + TT IT	134 (42/46 (+ 46 in ARM3)	12-wk supcombined REX and AEX	2x/wk, 120 min/session, AEX: 35 min, Interval cycling: 4 min, 60–80% HRmax/3 min on 35% HRmax, REX (large muscle groups): 35 min, circuit exercise; 60–80% 1RM; 8–12 reps, 3 sets	Usual care	134/232: (57.7%)	EX: 7/34 (20.6%) C: 3/46 (4.7%) in total 10/80 (12.5%) *n* = 8 did not receive EX	sessions average: 8 ± 3.5/24 Treatment integrity 86%	EX: muscle pain overuse (*n* = 4), cardiac complaints (*n* = 1), increased fatigue after training (*n* = 1)	none	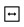 Fatigue (CIS and C30) 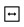 QoL (C30) 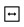 Physical function (C30) 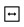 Emotional function (C30) 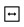 Functional impairments (C30)
Psyzora et al., 2017 [41]	Advanced cancer patients, admitted to palliative care (*n* = 15) Alimentary system, *n* = 13 urogential system, *n* = 8 mammary gland, *n* = 6 Hematologica, *n* = 6 indefinite origin, *n* = 5 lung, *n* = 5 central nervous system, *n* = 1 mouth, *n* = 1 skin)	Ex: 72.4 ± 9.5; C: 69.3 ± 13.7	n.a.	60 (30/30)	2-wk physiotherapeutic exercise, MFR, and PNF	3x/wk, 30 min, active exercise of upper and lower limbs, selected techniques of MFR and PNF	Usual care	n.a. *	EX: 1/30 (3.3%) C: 1/30 (3.3%) in total 2/60 (3.3%)	n.a. *	n.a. *	↑ Fatigue Severity (BFI and ESAS) (*p* < 0.01)	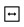 Depression (ESAS) 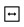 Anxiety (ESAS)
Rief et al., 2014 + [33,43]	Cancer patients with metastatic progress in thoracic/lumbar spine or in sacrum (*n* = 20 lung, *n* = 14 prostate, *n* = 11 breast, *n* = 3 renal, *n* = 2 melanoma, *n* = 10 other)	Ex: 61.3 ± 10.1; C: 64.1 ± 10.9	RTx	60 (30/30)	2-wk sup isometric REX followed by 12-wk home-based training	5x/wk, 30 min isometric spinal training of the autochthonous muscles + unsupervised 3x weekly home training	Usual care, aspiration exercise, hot roll treatments	60/80: (75%)	EX: 5/30 (16.6%) C: 7/30 (23.3%) in total 12/60 (20%)	patients completed the exercise protocol: 25/30 (83.3%)	n.a. *	↑ 30 s sit-to-stand (*p* < 0.001), ↑ QoL (BM22) (*p* = 0.01) ↑ Fatigue (BM22) (*p* = 0.01) ↑ Pain (VAS) (*p* = 0.003)	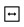 Functional interference (BM22) 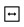 Emotional interference (BM22) 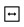 Cognitive interference (BM22) 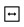 Overall survival 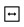 Progression-free survival
Tsianakas et al., 2017 [40]	Recurrent advancing or metastatic cancer: *n* = 15 prostate, *n* = 9 gynecological, *n* = 9 hematological, *n* = 7 breast, *n* = 5 colorectal, *n* = 1 upper gastrointestinal	Ex: 65 ± 11.7 C: 66.2 ± 10.2	n.a.	42 (21/21)	12-wk walking intervention	30 min walking on alternate days	n.a. *	42/110: (38.1%)	EX: 8/21 (38.1%) C: 7/21 (33.3%) in total 15/42 (35.7%)	n.a. *	0	none	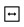 QoL (FACT-G) 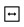 Global fatigue score (BFI)
Zhou et al., 2017 [37]	Advanced nasopharyngeal cancer stage III/IV	n.a. *	CTx RTx	114 (57/57)	Tai Chi exercise (24-form Yang style) n.a. duration	5x/wk 60 min (10 min. warm up, 30 min Tai Chi exercise, 10 min breath and mediation; 10 min relaxation)	Usual care	114/130: (87.6%)	EX: 15/57 (26.3%) C: 16/57 (28%) in total 31/114 (27.1%)	n.a. *	0	↑ Fatigue (MFSI-SF) (*p* < 0.05) ↑ General fatigue (MFSI-SF) (*p* < 0.05) ↑ Physical fatigue (MFSI-SF) (*p* < 0.05) ↑ Emotional fatigue [MFSI-SF] (*p* < 0.05)	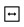 Mental fatigue (MFSI-SF)
Zimmer et al., 2017 [39]	Metastasized colorectal cancer	Ex: 68.5 C: 70	CTx + TT no treat TT	30 (17/13)	8-wk sup exercise, combining endurance, REX, and balance exercise	2x/wk, 60 min (Warmup 10 min. balance training, AEX 60–70% of HRmax, 20 min, REX 5 stations circuit 60–80% of h1RM, 8–12 reps, 2 sets 60–80%; (cool down)	Usual care	n.a. *	EX: 2/17 (11.7%) C: 4/13 (30.7%) in total 6/30 (20%)	Mean training frequency: 88.3%	0	8 wk: ↑ Trial Outcome Index (*p* = 0.028) ↑ Muscle strength (bench and leg press, lat pulldown) (*p* = 0.002) ↑ GGT_Reha static 2 (*p* < 0.025) 14 wk Follow-Up: ↑ Trial Outcome Index (*p* = 0.031) ↑ Muscle strength (bench and leg press, lat pulldown) (*p* < 0.05)	8 wk/14 wk: 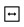 Physical Well-being (FACT/GOG-NTX) 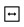 Functional Well-being (FACT/GOG-NTX) 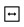 Social Well-being (FACT/GOG-NTX) 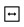 Emotional Well-being (FACT/GOG-NTX) 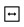 QoL (FACT-G)

Abbreviations: aerobic exercise (AEX), adverse event (AE), Ambulatory Post Acute Care Daily Activities Short Form (AM-PAC CAT), Ambulatory Post Acute Care Daily Mobility Short Form (AM-PAC Mobility), androgen-suppression therapy (AST), anticancer treatment (Treat), Big Five Inventory (BFI), control group (C), chemotherapy (CTx), EORTC QLQ-BM22 (BM22), EORTC QLQ-C30 Questionnaire (C-30), exercise group (EX), Functional Assessment of Cancer Therapy—Fatigue-Scale (FACT-F), Functional Assessment of Cancer Therapy—General (FACT-G), Six Minute Walk Test (6 MWT), Functional Assessment of Cancer Therapy/Gynecologic Oncology Group—Neurotoxicity (FACT-GOG-NTX), hormone therapy (HT) hypothetical one-repetition maximum (h1RM), immunotherapy (IT), one-repetition maximum (1RM), monoclonal antibody (MAB), maximum heart rate (HRmax), Multidimensional Fatigue Syndrome Inventory—Short Form (MFSI-SF), myofascial release (MFR), norm-based scoring (NBS), Orientation to Life Questionnaire (L-13), previous (Prev.), proprioceptive neuromuscular facilitation (PNF), radiation treatment (RTx), Remained stable ( 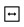), resistance exercise (REX), sensory organization test (SOT), supervised (sup), The Edmonton Symptom Assessment System (ESAS), Trial Outcome Index (TOI—Sum of physical well-being and functional well-being intended as summary index of functional status), surgery (SX), quality of life (QoL), weeks (wk), participants (pts), target therapy (TT), 36-Item Short Form Survey (SF-36). * n.a. = data not available in publication/data not shown, ∞ Erratum from 2015 correction to: Prostate Cancer and Prostatic Diseases (2013) 16, 328–335; doi:10.1038/pcan.2013.22. + Studies by Rief et al. from 2014 (a) and (b) are summarized.

**Table 2:** Study characteristics of controlled trials.

Author	Cancer Entities	Age	Treat	*n* (EX/C)	Description of Intervention	Exercise Modalities	Control	Recruitment	Drop-Out Rate:	Adherence	AE	EX vs. UC ↑ = Improvement; ↓ = Worsening;	EX vs. UC (Non Sign.)
Buss et al., 2009 [44]	Advanced cancer patients; short lifetime expectancy (approximately 1–2 months)	n.a. *	none	57 (38/19)	4-wk sup, individualized kinesiotherapy	3x/wk, 20–30 min	n.a.*	57/80 (71.2%)	EX: 12/38 (31.6%) C: 2/19 (10.5%) in total 14/57 (24.5%)	n.a. *	n.a. *	↑ Fatigue (BFI) wk 3 and wk 4 (*p* < 0.001) ↓ Intensification physical symptoms (RSCL) (*p* < 0.05)	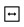 QoL 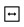 Mental symptoms
Zhao et al., 2015 [45]	Head and Neck squamous cell cancer, Stage III and IV (*n* = 14 oropharynx, *n* = 1 larynx, *n* = 1 nasopharynx, *n* = 2 unknown)	Ex: 57 ± 7 C: 57 ± 7	CTx	20 (11/9)	14-wk REX and walking exercise (1st 7 wk sup, 2nd 7 wk home-based)	3x/wk, 60 min, moderate intensity, REX: 8–12 reps, 3 sets lking EX: 30 min per wk, Home-based exercise individualized but also combined REX and walking exercise	Usual care	20/27: (74.1%)	EX: 1/11 (9.0%) C:2/9 (22.2%) in total 3/20 (15%)	mean session: 15.2/21; attendance rate: 72% home- based training adherence n.a.	0	7 wk: ↑Vitality/Fatigue (SF36) (*p* < 0.05) ↑Mental Well-being (SF36) (*p* < 0.05) 14 wk: ↑ strength knee extension (*p* < 0.05) ↑ Mental Well-being (SF36) (*p* < 0.05)	7 wk: 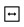 6 MWT 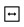 BMI 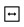 QoL [SF36] 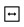 Physical activity 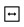 Lean body mass 14 wk: 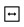 6 MWT 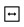 BMI 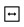 QoL [SF36] 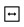 Physical activity 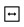 Vitality/Fatigue [SF36) 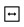 Lean body mass

Abbreviations: adverse event (AE), anticancer treatment (Treat), Big Five Inventory (BFI), body mass index (BMI), control group (C), chemotherapy (CTx), exercise group (EX), quality of life (QoL), Short Form 36 (SF-36), Remained stable ( 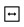), resistance exercise (REX), Six Minute Walk Test (6 MWT), supervised (sup), The Rotterdam Symptom Checklist (RSCL); week (wk). * n.a. = data not available in publication/ data not shown.

**Table 3:** Drop-outs with reasons.

Drop-Out	In Total *n*	Exercise *n*	Control *n*
Participants	220 (100%)	128 (58.7%)	92 (41.8%)
Died due to cancer	61 (27.7%)	39 (30.5%)	24 (26.0%)
Disease progression/health deterioration	113	68	45
	(51.3%)	(53.1%)	(48.9%)
Lost to follow-up/ non-compliant	35 (15.9%)	17 (13.3%)	19.5 (20%)
Various reasons *	6 (2.7%)	1 (0.8%)	5 (5.4%)
Adverse Events *^1^	3 (1.3%)	3 (2.3%)	0 (0%)

Ex = exercise group; C = control group; * various reasons can be lack of time, too far to travel, or appointment burdens, etc. *^1^ In total, 9 adverse events occurred, but only 3 led to drop-out.

## Data Availability

Publicly available datasets were analyzed in this study. This data can be found in the cited publications from reference [31,32,33,34,35,36,37,38,39,40,41,42,43,44,45].
